# Generation and characterization of a novel transgenic mouse harboring conditional nuclear factor-kappa B/RelA knockout alleles

**DOI:** 10.1186/s12861-016-0135-8

**Published:** 2016-09-23

**Authors:** Talha Ijaz, Maki Wakamiya, Hong Sun, Adrian Recinos, Ronald G. Tilton, Allan R. Brasier

**Affiliations:** 1 Departments of Biochemistry and Molecular Biology, University of Texas Medical Branch, Galveston, TX USA; 2 Institute for Translational Sciences, University of Texas Medical Branch, Galveston, TX USA; 3 Internal Medicine-Division of Endocrinology, University of Texas Medical Branch, Galveston, TX USA; 4 Sealy Center for Molecular Medicine, University of Texas Medical Branch, MRB 8.128, 301 University Blvd, Galveston, TX 77555-1060 USA

**Keywords:** NF-kB, RelA, p65, Flox, Tamoxifen, Cre, Col1a2

## Abstract

**Background:**

Nuclear Factor-Kappa B (NF-kB) is a family of transcription factors that are important in embryonic development, inflammation, epithelial-to-mesenchymal transition and cancer. The 65 kDa RelA subunit is the major transcriptional activator of the NF-kB pathways. Whole-body deficiency of RelA leads to massive apoptosis of liver hepatocytes and death *in utero*. To study the role of RelA in physiology and in disease states in a manner that circumvents this embryonic lethal phenotype, we have generated a mouse with RelA conditional knockout (CKO) alleles containing *loxP* sites that are deleted by activated Cre recombinase.

**Results:**

We demonstrate that RelA^CKO/CKO^ mice are fertile, do not display any developmental defects and can be crossed with Cre-expressing mice to delete RelA in a temporal, tissue-specific manner. Our mating of RelA^CKO/CKO^ mice with Zp3-Cre transgenic led to embryonic lethality of RelA-deficient embryos. In contrast, mating of RelA^CKO/CKO^ mice with Col1α2-CreER mice allowed for the generation of double transgenics which could be stimulated with tamoxifen to induce fibroblast-specific RelA deletion in adulthood.

**Conclusions:**

Based on our collective data, we conclude that this novel RelA^CKO/CKO^ mouse allows for efficient deletion of RelA in a tissue-specific manner. This RelA^CKO/CKO^ mouse will be an invaluable tool for deciphering the mechanistic roles of RelA in various cells and tissues during development and in disease.

**Electronic supplementary material:**

The online version of this article (doi:10.1186/s12861-016-0135-8) contains supplementary material, which is available to authorized users.

## Background

NF-kB is an inducible transcription factor complex involved in the regulation of genes necessary for cell survival, differentiation, immunity and inflammation [1]. RelA, C-Rel and RelB are transcriptional activating subunits that contain a N-terminal Rel homology domain (RHD) and a C-terminal transcription activation domain (TAD) whereas NF-kB1 (p50/p105) and NF-kB2 (p52/p100) are DNA-binding proteins that contain C-terminal autoinhibitory ankyrin repeat domains [2]. In most cells, NF-kB is high molecular weight heterodimeric complexes containing a DNA-binding- and a transcription activator subunit retained in the cytoplasm by Inhibitor of kappa B (IkB) proteins. Receptor activation by numerous ligands induces phosphorylation of IkBα, targeting it for degradation and allowing nuclear translocation of RelA•NF-kB1 dimers, the most abundant and most potent transcription factor pair required for activation of inflammatory and anti-apoptotic gene expression programs [3].

Beg and colleagues were the first to describe the lethality of whole-body RelA deficiency in mice [4]. Mating of RelA heterozygous mice failed to generate any RelA-deficient (RelA^-/-^) animals and an analysis of embryos indicated that RelA^-/-^ embryos undergo massive liver degeneration with hepatocyte apoptosis and consequently die *in utero*. These findings were independently corroborated using a different gene-targeting vector [5]. This study demonstrated that when RelA^-/-^ embryonic livers are transplanted into SCID mice, T and B cells populate spleen and lymph nodes to similar extent as wild-type (WT) cells, suggesting that the lethal effect of RelA^-/-^*in utero* is due to impairment of hepatocyte development and not due to deranged hematopoiesis. In a subsequent finding, the lethal phenotype of RelA^-/-^ was rescued by absence of TNFα [6]. RelA activation downstream of the TNF-receptor is necessary for the production of anti-apoptotic molecules, including TRAF-1, TRAF-2, c-IAP1 AND c-IAP2 that protect cells from local, endogenously produced TNFα-induced apoptosis [7, 8].

To overcome embryonic lethality produced by whole-body RelA deletion and to investigate the role of RelA in disease-specific states, investigators have generated transgenic mice inserting *loxP* sites in the *RelA* gene (RelA-floxed). DNA recombination mediated by Cre recombinase in RelA-floxed animals leads to truncation or deletion of the RelA protein. Algul and colleagues created a RelA-floxed mouse containing *loxP* sites in *RelA* introns 6 and 10 [9]. Induction of Cre led to deletion of exons 7–10, encoding the RHD, producing a truncated RelA that failed to translocate to the nucleus. This strategy results in expression of a truncated RelA C-terminal TAD that potentially confounds the study. Recently, another group created RelA-floxed mice with *loxP* sites flanking the promoter region and exon 1 that resulted in the deletion of RelA in B-cells when RelA-floxed animals were crossed with CD19-Cre trasngenics [10]. The strategy to excise only exon 1 of the RelA gene has the potential to allow the expression of alternatively spliced variants. In the case of RelA, there is putative internal ribosome entry site (IRES) in exons 4–5 which allows for 5′cap-indepedant translation [11]. Additionally, there is evidence of alternatively spliced variants of RelA in different cell types. For example, p65Δ variant lacks aa 222–231 and is abundantly expressed in pre-B and erythroid colony forming cells [12]. During screening of human adult osteoblastic cDNA library a RelA variant, p65Δ2, has been found that lacks amino acids (aa) 13–25 and 506 [13]. The N-terminus aa 13–25 are part of the RHD, corresponding to exon 2 and 3, and may be needed for binding to DNA. Although there has not been any exhaustive investigation into alternative RelA splicing variants, the presence of IRES in exons 4–5 and existence of RelA NH2-terminal splice variants suggests that some RelA transcripts may be produced in specialized cells under pathophysiological conditions that lack exon 1 but still can undergo translation. These splice variants would not be targeted in the flox mouse created by Heise and colleagues. Furthermore, cells that undergo recombination in the transgenic by Hesie et al. begin expressing eGFP. This strategy makes it easier to track recombined cells but the eGFP signal could be confounding in leukocytes, such as monocytes/macrophages, that have significant intracellular granules and are autofluorescent in the same emission range as GFP [14]. We decided to create a RelA-floxed mouse for investigating NF-kB signaling in vivo because, at the time, no such experimental tool was commercially available. Our group has developed a RelA-floxed transgenic mouse that allows for complete deletion of RelA and which will be made available for use by the scientific community.

**Fig. 1:**
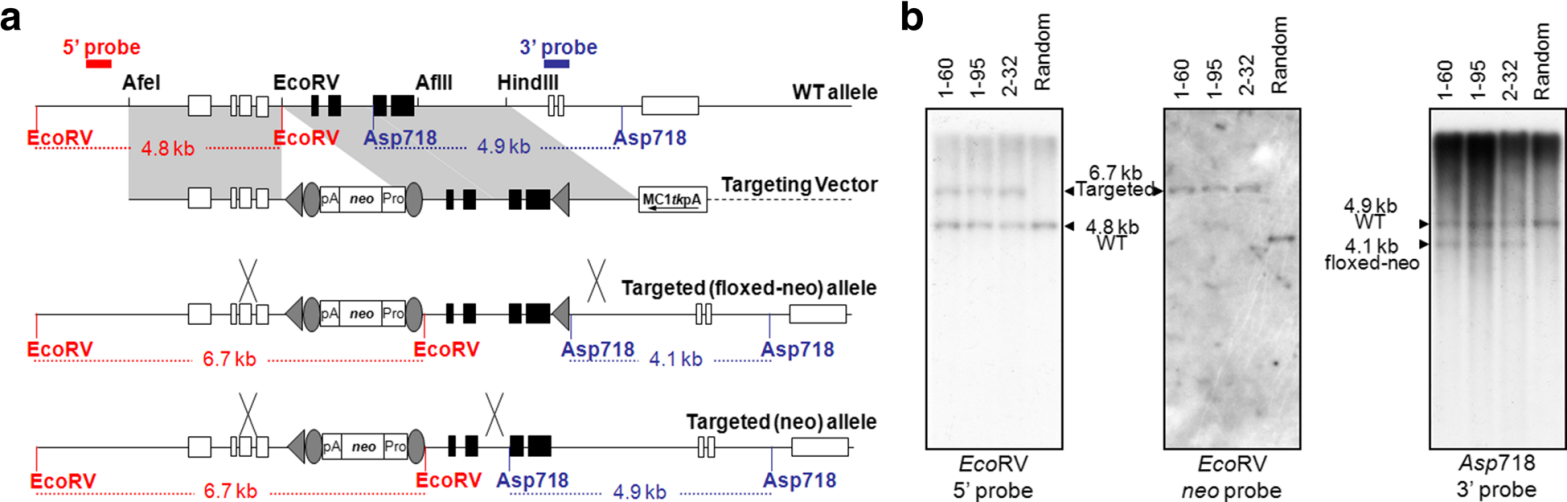
Strategy for targeted mutation of the *RelA* gene. **a** The open boxes represent exons 1–4, and 9–11; the solid boxes represent exons 5–8. The solid lines represent intronic sequences; the dashed line represents plasmid vector DNA. The triangles represent *loxP* sequences and the ovals represent *frt* sequences. The gene-targeting vector possesses a total of 7.2-kb homologous sequence. The PGK*neo*bpA cassette was inserted into an *Eco*RV site located ~0.6-kb upstream of exon 5. The second (stand-alone) *loxP* was inserted into an *Afl*II site located ~240-bp downstream of exon 8. The vector has a 2.9-kb 5′-homology arm, and a 3′-homology arm divided into 2.7-kb and 1.7-kb segments. Homologous recombination between WT *RelA* and the targeting vector may result in a *RelA* mutant allele that carries both PGK*neo*bpA cassette and the stand-alone *loxP* [Targeted (floxed-neo) allele], or one that carries only PGK*neo*bpA cassette [Targeted (neo) allele]. The 5′-flanking probe hybridized to *Eco*RV-digested genomic DNA detects 4.8-kb WT bands, and 6.7-kb targeted allele bands; the 3′-flanking probe hybridized to *Asp*718-digested genomic DNA detects 4.9-kb WT bands, 4.1-kb targeted (floxed-neo) allele bands, and 4.9-kb targeted (neo) allele bands. **b** Southern blot analysis of DNA isolated from ES cell clones. Three clones, 1–60, 1–95, and 2–32, were found correctly targeted in the initial screening, and further expanded; DNA was isolated and subjected to Southern blot analysis. DNA isolated from a random integration clone was used as a non-recombinant control (Random). A restriction enzyme and a probe used in the analysis were indicated below each panel

The mouse *RelA* gene, comprised of 11 exons, is located within a 10.65-kb region on Chromosome 19. Our strategy was to introduce two *loxP* sites into intron 4 and intron 8 of the *RelA* gene via gene targeting [*RelA conditional knockout* (*RelA*^*CKO*^) allele, Fig. 1]. In the presence of Cre recombinase, a 2.7-kb sequence containing exons 5–8 would be excised from RelA^CKO/CKO^ generating the RelA^-/-^. The deletion of exons 5–8, which encode the C-terminus of its RHD (aa 113–292), results in a downstream frame-shift that will further lead to degradation of RelA transcripts via nonsense-mediated mRNA decay.

## Results and Discussion

**Fig. 2:**
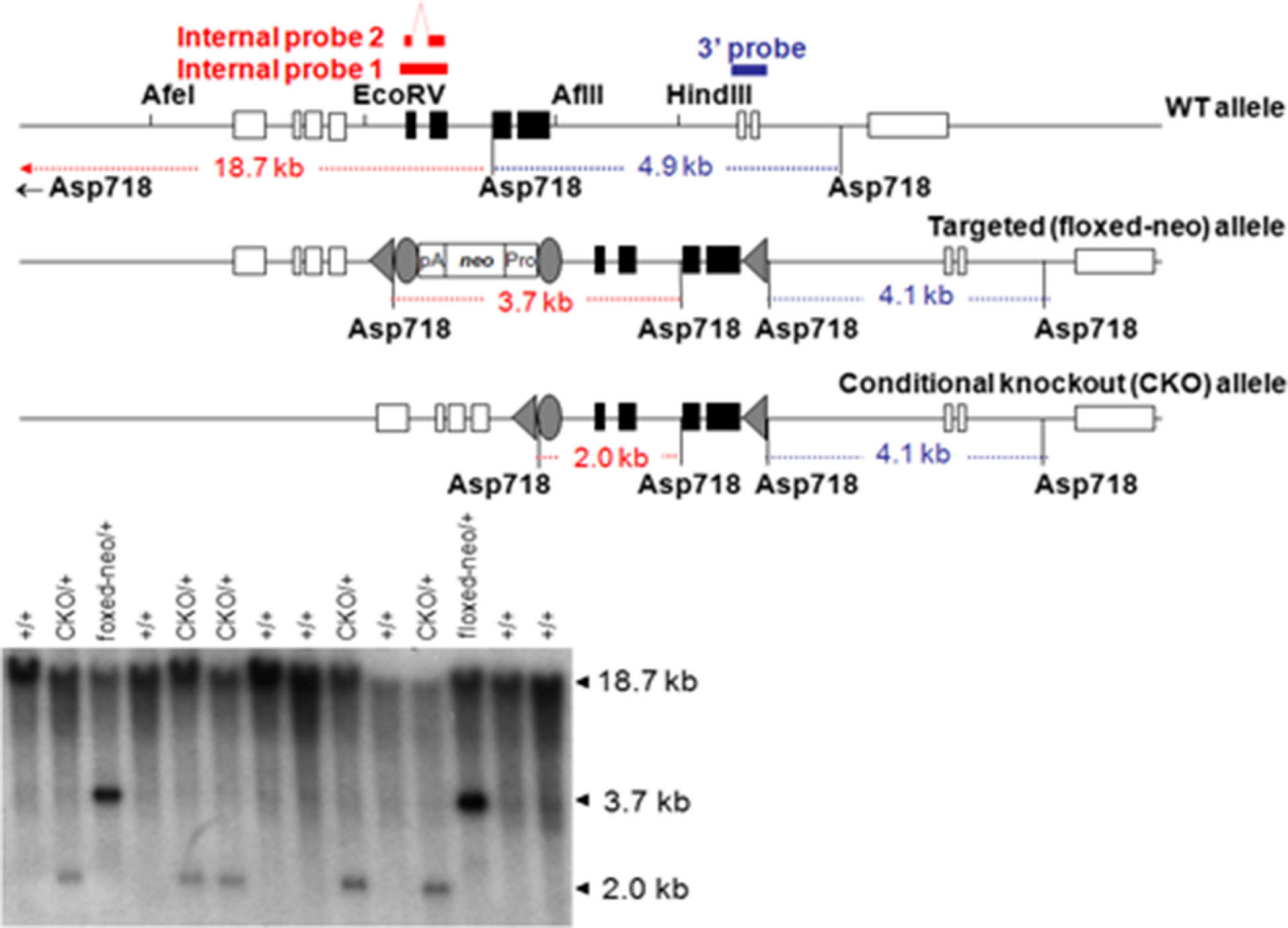
Confirmation of PGK*neo*bpA cassette removal. The internal probes hybridized to *Asp*718-digested genomic DNA detect 18.7-kb WT bands, 3.7-kb targeted (floxed-neo) allele bands, and 2.0-kb CKO allele bands. Tail DNA was isolated from pups generated by mosaic females (*RelA* ^*floxed-neo/+*^ ↔ *RelA* ^*CKO/+*^; *Gt(ROSA)26Sor* ^*FLP1/+*^) and a wild-type C57BL/6J male, and digested by *Asp*718. The blot was hybridized with the internal probe 1. When the same filter was stripped and rehybridized with the 3′ probe, all CKO/+ and neo/+ samples showed 4.1-kb mutant bands and 4.9-kb WT bands (data not shown)

The gene-targeting vector containing an *frt*-flanked neomycin positive-selection cassette, and a thymidine kinase negative-selection cassette (see Methods and Fig. 1) was electroporated into 129B6F1 hybrid mouse embryonic stem (ES) cells, G4 [15]. One hundred-forty ES cell colonies survived 9-days of double-drug selection. Colonies were selected and propagated and DNA isolated from the cells was screened for homologous recombinants by Southern blotting. We found that twelve clones underwent homologous recombination at the *RelA* locus, and of those, three clones incorporated a “stand-alone” *loxP* sequence in intron 8 (Fig. 1a). After further expansion and repeated Southern blot analysis (Fig. 1b), all three clones were injected into C57BL/6J blastocysts. We then bred the resulting male chimeras with FLPeR mice on a C57BL/6J background [B6.129S4-*Gt(ROSA)26Sor*^*tm1(FLP1)Dym*^/RainJ, the Jackson Laboratory, Stock Number: 009086] to obtain offspring double-heterozygous for *RelA*^*CKO*^ allele and *FLP1* knock-in allele. The FLPeR mice express *FLP1* recombinase in a wide range of cell types including germ cells [16], and were used for FLP-mediated removal of the neomycin cassette from the mutant *RelA* allele (Fig. 2). Germ-line transmission of *RelA* mutation was achieved with two clones, 1–60 and 1–95. We then backcrossed double-heterozygous mice to C57BL/6J mice to segregate the *FLP1* transgene and to establish RelA^CKO^ lines. Mice homozygous for *RelA*^*CKO*^ allele (RelA^CKO/CKO^) were normal, fertile and expressed RelA at normal levels.

**Fig. 3:**
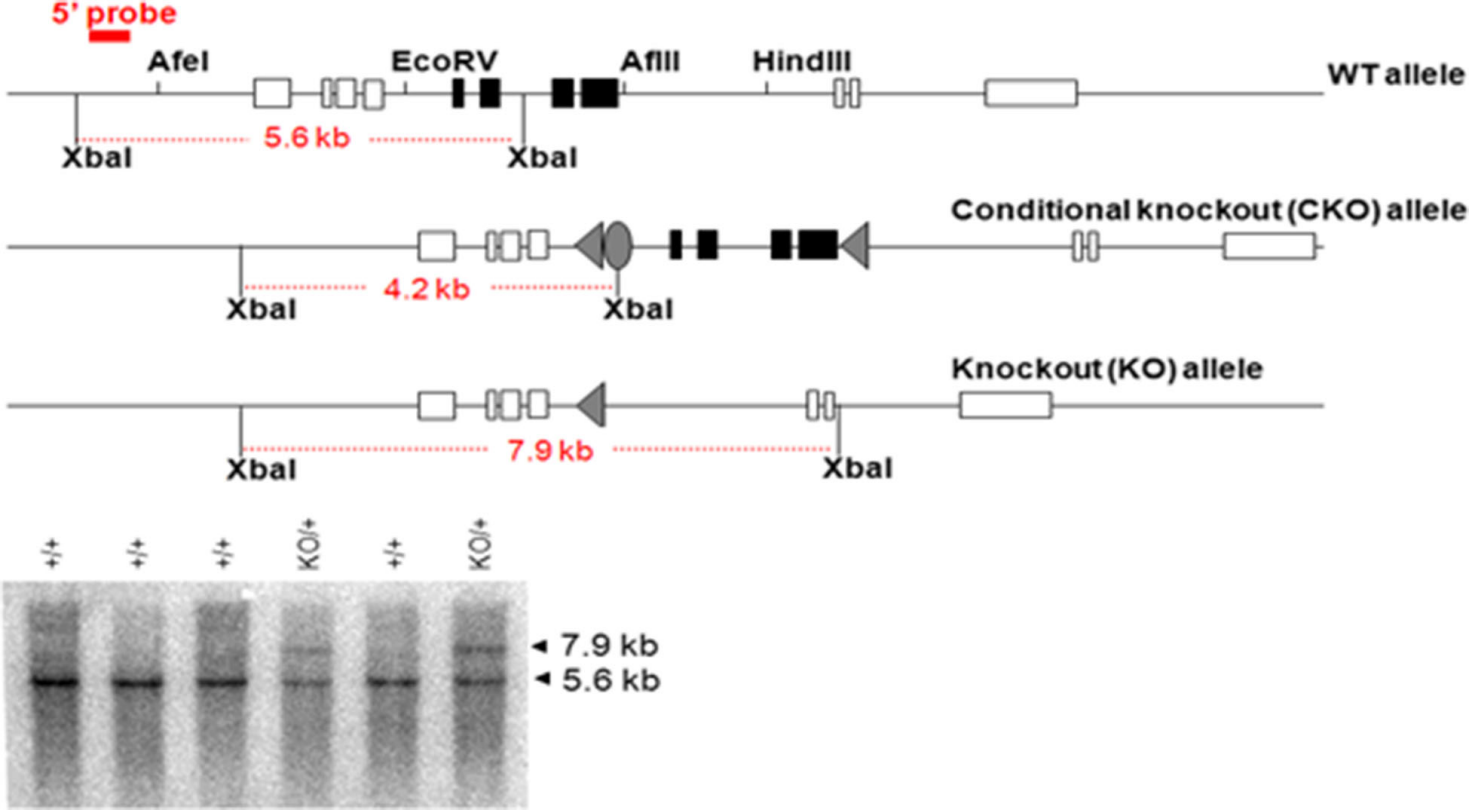
Confirmation of exon-5-8 removal. The 5′ probe hybridized to *Xba*I-digested genomic DNA detects 5.6-kb WT bands, 4.2-kb CKO allele bands, and 7.9-kb KO allele bands. Tail DNA shown in the figure was isolated from pups generated by a female heterozygous for *RelA* ^*CKO*^ allele and hemizygous for Zp3-cre transgene, and a wild-type C57BL/6J male

**Fig. 4:**
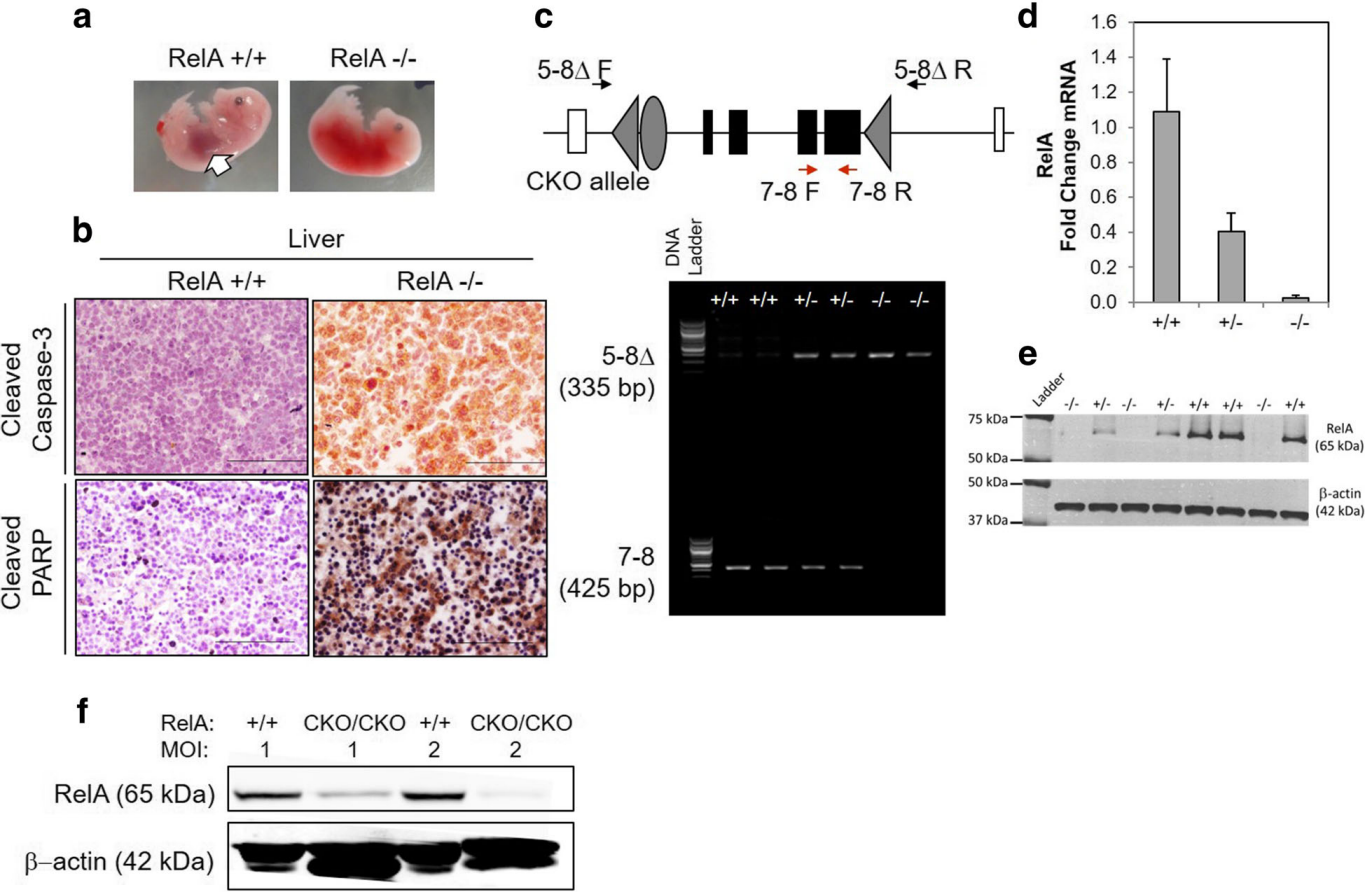
Characterization of whole-body RelA^-/-^ and RelA^CKO/CKO^ MEF cells. **a** Mating of a RelA^+/-^ male and female mice produced RelA^+/+^, RelA^+/-^ and RelA^-/-^ embryos that were isolated on E12. White arrow indicates the liver, which was observed macroscopically in RelA^+/+^ and RelA^+/-^ embryos only, whereas a large hematoma was observed in abdomens of RelA^-/-^ embryos. **b** Immunohistochemistry for cleaved-caspase 3 and cleaved-PARP demonstrated paucity of immunostaining in WT embryos for but very robust staining for the enzymes in KO embryos indicative of massive liver apoptosis. **c** PCR was performed on DNA isolated from the embryos. Presence of the 7–8 product (425 bp) indicates presence of a WT allele whereas presence of the 5–8Δ product (335 bp) indicates a RelA mutant allele. **d** QRT-PCR on RNA isolated from MEF cells provides evidence that RelA transcripts decreased with decrease in RelA WT allele. **e** Western blot of proteins extracted from the same MEF cells probed with anti-RelA Ab (top). β actin staining was used as loading control (bottom). There was no shift in the RelA band suggesting absence of any truncated form of RelA. These data suggest decrease in the RelA protein with decreasing dose of WT RelA DNA. **f** RelA^CKO/CKO^ MEF cells were transduced with lentiviral-Cre at multiplicity of infection (MOI) of 1 and 2. Induction of Cre led to a substantial decrease in RelA with increasing MOI

**Fig. 5:**
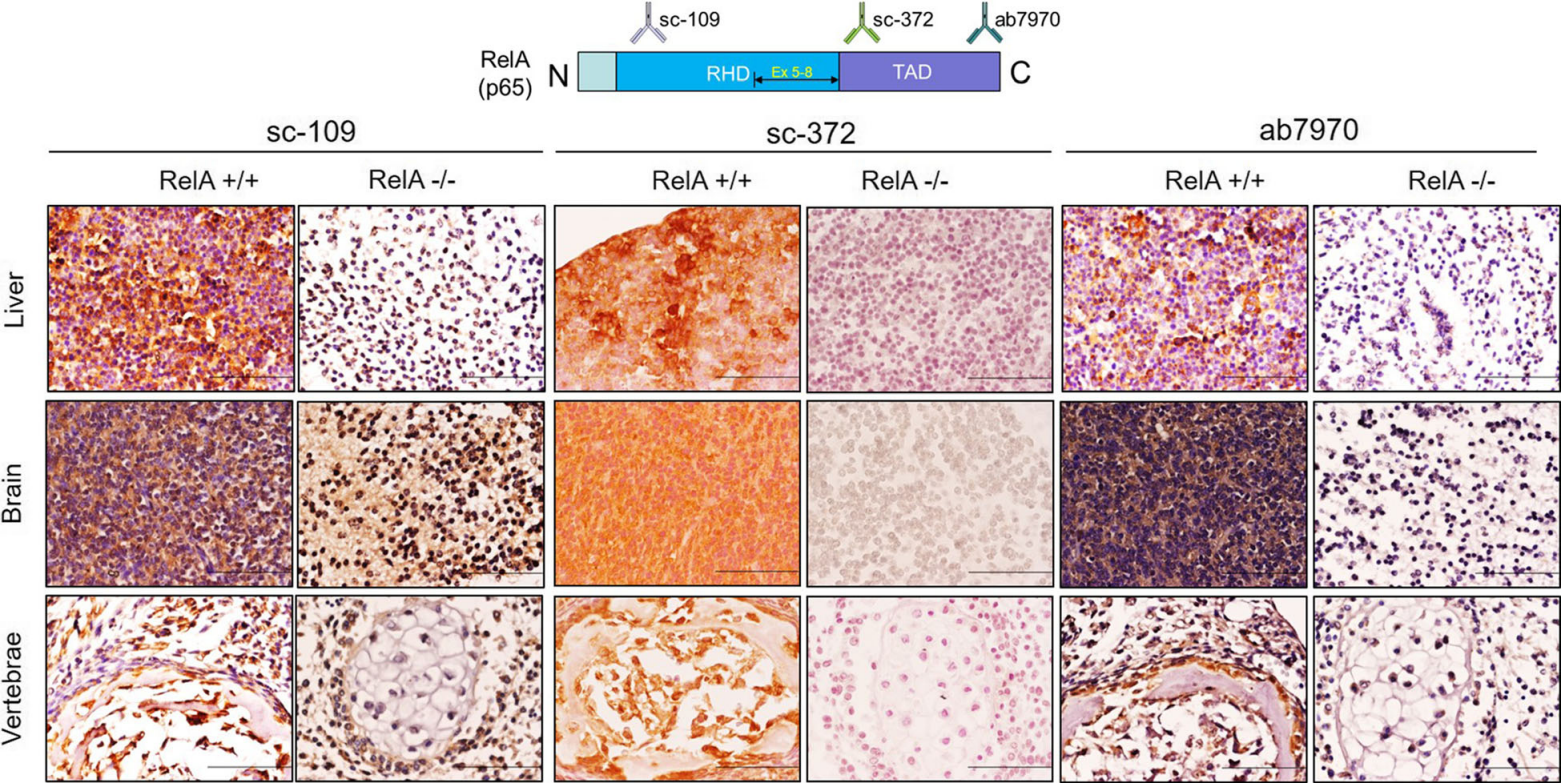
Verification of RelA deletion via immunohistochemistry using antibodies targeting RelA N- and C-terminus. RelA^+/+^ and RelA^-/-^ embryos, identified by PCR screen, were sectioned and immunostained for RelA with three different antibodies targeting different regions of RelA as depicted in the diagram. Robust immunostaining of RelA was observed with all three antibodies in the developing liver, brain and vertebrae of RelA^+/+^ embryos. In contrast, there was a complete absence of signal in RelA^-/-^ embryos with antibodies targeting the C-terminus and a slight signal was observed in the liver with antibody targeting the N-terminus, possibly due to antibody cross-reactivity with other RHD containing proteins. This suggests that the recombined RelA alleles fail to produce RelA protein. *N* = 3 RelA^+/+^ and *n* = 3 Rel^-/-^ embryo tissue sections were used in each experiment. Representative images from three experiments are shown. All images were captured at 600× magnification. Scale bar represents 50 microns

To generate whole-body RelA deficient mice containing the knockout alleles [KO, Fig. 3], we first crossed a RelA^CKO/+^ mouse (1–60 line) and a Zp3-cre transgenic mouse on a C57BL/6 background [C57BL/6J-Tg(Zp3-cre)93Knw/J, The Jackson Laboratory, Stock Number: 003651, [17]], and obtained a RelA^CKO/+^; Tg(Zp3-cre)93Knw/0 female mouse. In Zp3-cre transgenic mice, Cre expression is controlled by regulatory sequences from the mouse *zona pellucida 3 (Zp3)* gene, which is normally expressed exclusively in the growing oocytes, and Cre-mediated *loxP* recombination occurs in 100 % of oocytes [18]. Therefore, backcrossing the RelA^CKO/+^; Tg(Zp3-cre)93Knw/0 female mouse to a wild-type C57BL/6J male results in offspring: RelA^+/-^; Tg(Zp3-cre)93Knw/0, RelA^+/+^; Tg(Zp3-cre)93Knw/0, RelA^+/-^ and RelA^+/+^. The offspring were genotyped by Southern blot (Fig. 3), and a RelA^+/-^ mouse was backcrossed to C57BL/6J mice for the propagation of the KO line. RelA^+/-^ male and female mating produced only RelA^+/-^ and RelA^+/+^ embryos. Analysis of embryos at embryonic day 12 (E12) demonstrated that almost a quarter of them had intraabdominal hemorrhages with no clear macroscopic discernment of a liver (Fig. 4a). Genotyping verified that these embryos were RelA^-/-^. Immunohistochemical analysis indicated very high immunoreactivity for cleaved-caspase 3 in the liver, signifying aberrant apoptosis (Fig. 4b). To further verify caspase-3 activation and apoptosis, we probed for cleaved poly ADP-ribose polymerase (PARP)-1, a protein that is a target of activated caspase-3 in its latent form. RelA^-/-^ embryonic liver had robust immunostaining of cleaved PARP-1 which was absent in RelA^+/+^ livers. Isolated mouse embryonic fibroblasts (MEFs) were used to verify DNA recombination and RelA mRNA and protein depletion in RelA^+/-^ and RelA^-/-^ cells (Fig. 4c–e). We failed to observe any shift in the RelA band (65 kDa) using the Santa-Cruz antibody sc-372 that targets the TAD, suggesting that a truncated RelA is not produced in the genetically recombined cells. Furthermore, immunohistochemical analysis was performed using three different antibodies targeting the N- and C-terminus domains of the RelA protein (Fig. 5). All three antibodies demonstrated robust RelA immunostaining in the liver, brain and vertebrae of RelA^+/+^ embryos. Two antibodies targeting the C-terminus failed to show any staining in the RelA^-/-^ embryos whereas slight staining was observed in the liver with sc-109 antibody that recognizes the RHD at the N-terminus. The minor staining could be due to antibody cross-reactivity with other RHD-containing proteins such as RelB or C-Rel. To verify that the antibodies specifically recognized RelA, whole cell lysates from A549 cell line were immunoprecipitated with the three RelA antibodies and subjected to selected reaction monitoring- mass spectrometry analysis (SID-SRM-MS). Relative to IgG immunoprecipitates, there was 100-fold increase in RelA when immunoprecipitation was performed with sc-109 and ab7970 antibodies and approximately a 200-fold increase in RelA when sc-372 antibody was used (Additional file 1: Figure S1). We interpret this to mean that all three antibodies recognize RelA and RelA^CKO/CKO^ cells that have undergone DNA recombination at the RelA alleles do not produce RelA. To determine whether Cre-mediated recombination can also occur in vitro, we transduced Cre via a lentivirus vector in RelA^CKO/CKO^ MEFs. In experiments infecting MEFs with only a multiplicity of infection (MOI) of 2, we observed a 90 % reduction in RelA protein (Fig. 4f) with no shift in the RelA band. These data confirm that RelA can be depleted both in vitro using RelA^CKO/CKO^ cells and in vivo using our RelA ^CKO/CKO^ mice.

**Fig. 6:**
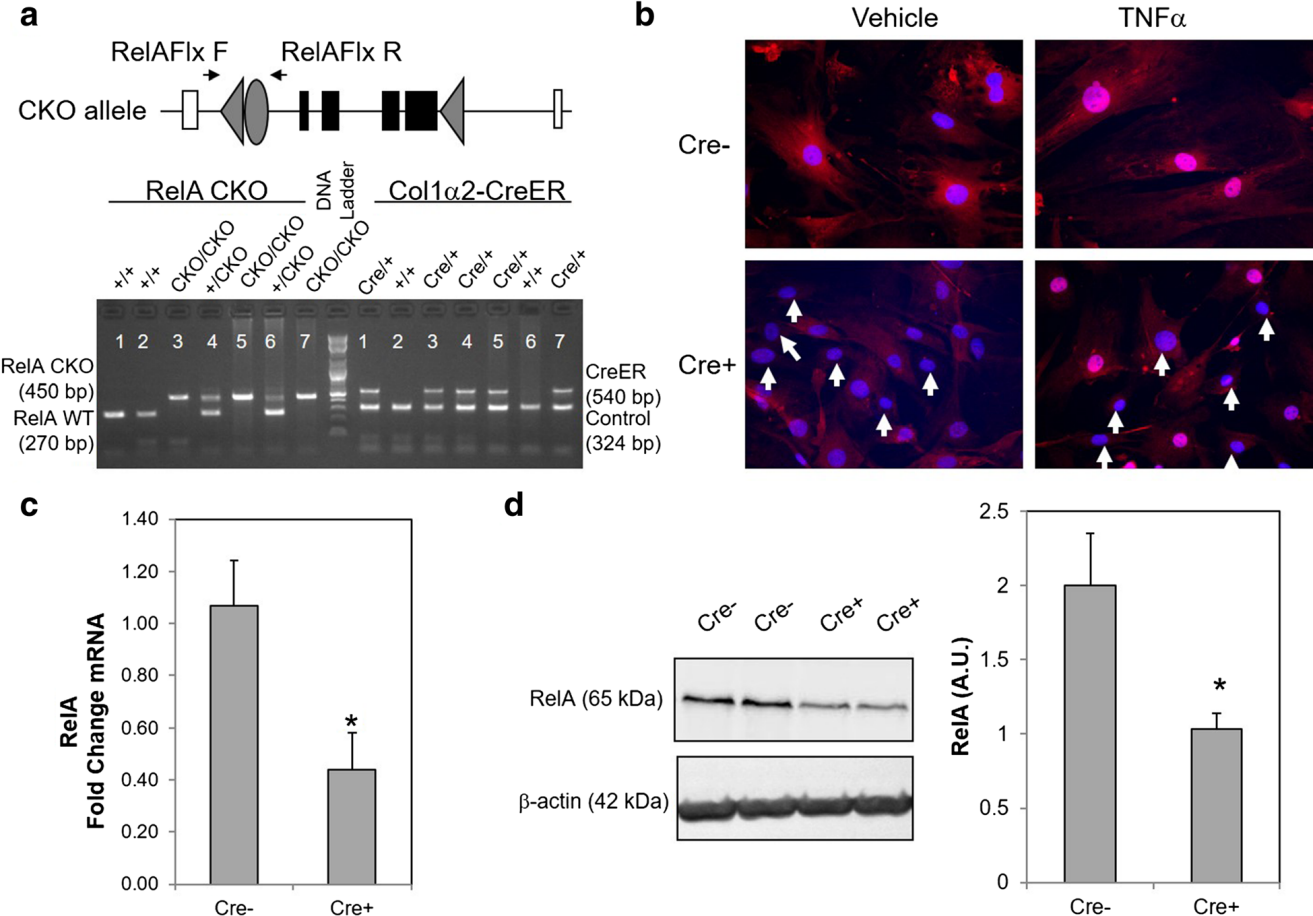
Tamoxifen mediated RelA deletion in skin fibroblast in vivo. **a** To generate fibroblast-specific RelA-/- mice, RelA CKO/CKO mice were crossed with Col1α2-CreER mice. PCR on DNA from tail biopsies was used to identify the double transgenic. Mice harboring the CKO allele were identified by the 450 bp product while those containing the WT allele were identified by the 270 bp product. **b** RelA^CKO/CKO^ Cre- and RelA ^CKO/CKO^ Cre + littermates were injected with 1 mg tamoxifen i.p. for 10 days. After 3 weeks, dermal fibroblasts were harvested from both genotypes and immunostained for RelA after stimulation with vehicle or TNFα (20 ng/ml). Cre + fibroblast had very little cytoplasmic RelA under basal condition and also displayed less accumulation of RelA in the nucleus after TNFα treatment. White arrows indicate cells that have decreased levels or absence of nuclear RelA. **c** QRT-PCR on fibroblasts isolated from Cre + (*n* = 4) and Cre- (*n* = 4) mice after tamoxifen treatment suggests significant decrease in RelA mRNA levels in Cre + fibroblasts. Data are represented as mean +/- SEM, *P < 0.04. **d** In addition, the same Cre + fibroblasts produce significantly less RelA protein as determined by Western blot. Data are represented as mean +/- SEM *P < 0.04. A.U is arbitrary units

Our ultimate goal is to study the tissue-specific role of RelA in vivo. We have previously demonstrated that aortic fibroblasts produce significant amount of IL-6 and MCP-1 that are NF-kB-dependent proteins, in vitro and the secretion of these cytokine/chemokine increases more than 2-fold in presence of monocytes [19, 20]. We have defined this interaction to be occurring in vivo in the aortic wall and contributing to aortic inflammation and to the development of aortic dissections [19]. To test the hypothesis that fibroblast-RelA contributes to aortic inflammation, we have developed fibroblast-specific RelA^-/-^ mice by crossing a tamoxifen inducible Col1α2-promoter driven Cre mouse (Col1α2-CreER) with RelA^CKO/CKO^ mice. Although the characterization of aortic fibroblast-RelA depletion and the role of fibroblast-RelA in aortic inflammation are currently being investigated by our lab, we have described here the experiments validating RelA depletion that also occurs in skin fibroblast cells. After three generations of crosses, RelA^CKO/CKO^ Cre + and RelA^CKO/CKO^ Cre- littermates (Fig. 6a), which appeared normal and fertile, were treated with tamoxifen and their skin fibroblasts were isolated for characterization. Cre + fibroblasts had decreased levels of RelA in their cytoplasm (white arrows in Fig. 6b) and in response to TNFα stimulation, failed to accumulate RelA in the nucleus. We observed that only 50 % of the fibroblast had decreased RelA immunostaining suggesting that tamoxifen administration affected only half of the skin fibroblasts. Furthermore, RelA transcripts (Fig. 6c) and protein (Fig. 6d) were depleted by approximately 50 % in Cre + fibroblasts. Collectively, these data demonstrated that Col1α2-CreER is efficiently activated in half of the dermal fibroblasts by tamoxifen administration and can promote the recombination of RelA alleles in these cells leading to RelA depletion. Our future work will utilize this transgenic mouse to explore the role of fibroblast-RelA in the development of vascular inflammation and fibrosis in vivo.

## Conclusion

In summary, we report the generation of a transgenic mouse containing RelA-flox alleles that recombine in the presence of active Cre recombinase to generate RelA^-/-^ cells. This will be an important tool for studies investigating RelA signaling in adult tissues since it allows one to bypass a major limitation, death *in utero*, that accompanies whole-body RelA^-/-^. Furthermore, this will be a valuable mouse model for exploring the role of RelA in development, an under-explored area due to a lack of appropriate tools for investigation.

## Methods

### Construction of the RelA conditional knockout vector

A 7.2-kb AfeI-HindIII DNA fragment (*RelA* 5′ upstream–intron 8 region) was isolated from a bacterial artificial chromosome (BAC) clone, RP24-329M16 (obtained from the Children’s Hospital Oakland Research Institute), and subcloned into pBluescript II SK (-) (Agilent, Santa Clara, CA) between *Sma*I and *Hind*III sites. A PGK*neo*bpA cassette [21] flanked by an *frt* site on one end and an *frt-loxP* sequence on the other was inserted into the unique *Eco*RV site in intron 4 in reverse orientation relative to the orientation of *RelA* transcription via blunt-end ligation*.* Oligonucleotides for creating a DNA fragment containing a *loxP* site with an inserted *Asp*718 site and two *Afl*II ends were synthesized and annealed, and inserted into a unique *Afl*II site in intron 8. Finally, MC1*tk*pA cassette was added into the *Sal*I site of the multiple cloning site region of the pBluescript for the enrichment of homologous recombinants via negative selection [22].

### Genetic engineering of mouse embryonic stem (ES) cells

The targeting vector was propagated, purified by cesium banding, and linearized by *Not*I. A total of 1 × 10^7^ cells suspended in DPBS was electroporated with 25 μg DNA using Gene Pulser (500 μF/230 V, BIO-RAD, Hercules, CA). The cells were then cultured in 200 μg/ml Geneticin (G418) and 200 nM 1-(2-deoxy-2-fluoro-1-D-arabinofuranosyl)-5-iodouracil (FIAU) for 9 days. ES cell colonies that survived the G418-FIAU drug selection were expanded in three sets of 96-well plates. One set of the cells was cryopreserved for blastocyst injection; the other two sets were processed for Southern blot analysis. The 5′ and 3′ flanking probes were hybridized to *Eco*RV-digested DNA and *Asp*718-digested DNA, respectively.

### Chimera production and animals

Correctly targeted ES cell clones were injected into C57BL/6J blastocysts and injected embryos were transferred to 2.5-day-post-coitum pseudo-pregnant Swiss Webster female mice. All animal work was approved by UTMB’s IACUC committee.

### Genotyping of the mouse using genomic Southern blot

Mice were genotyped by Southern blot analysis of tail DNA with 5′-flanking, 3′-flanking, and/or internal probes. All the probe fragments were generated by PCR, and subcloned into a T-vector. 5′-flanking probe: A 529-bp DNA was amplified form the BAC DNA with forward primers, TTGTGGGTAGCTGTGGTCAA, and reverse primer, CCAGCACTCCAGAAGAAAGG. 3′-flanking probe: A 468-bp DNA was amplified from the BAC DNA with forward primers, GGGAGAAGTGCAGCCCGGC, and reverse primer, CCCGGCCTCCCCCTGAGAA. Internal probe 1: A 561-bp DNA was amplified from the BAC DNA with forward primers, GATCCAGTGTGTGAAGAAGC, and reverse primer, GGTTATCAAAAATCGGATGT. Internal probe 2: A 209-bp DNA was amplified from a pool of cDNA originating from mouse embryonic fibroblast RNA with forward primers, GATCCAGTGTGTGAAGAAGC, and reverse primer, GGTTATCAAAAATCGGATGT. Genomic DNA digested by appropriate restriction enzymes was separated on agarose/TAE gels, and blotted onto Hybond-XL membranes (GE Healthcare, Waukesha, WI). Blots were hybridized at 65 °C overnight with radiolabeled and pre-associated probes in 1 M sodium chloride, 1 % SDS, 10 % dextran sulfate with 100 μg/ml salmon sperm DNA; and washed at 65 °C in 0.2X SSC, 0.1 % SDS.

### Generation of MEF cells and genotyping of the mouse using PCR

MEF cells were generated as described previously [23]. DNA was isolated from tail biopsies or MEF cells using phenol:chloroform extraction and ethanol precipitation followed by PCR. Briefly, tail biopsies or 500K cells were incubated with 700 μl of digestion buffer (50 mM Tris-HCl, pH 8; 100 mM EDTA; 100 mM NaCl; 1 % SDS; 350 μg Proteinase K) overnight at 55 °C. Next morning, 700 μl phenol:cholorform (Sigma) was added to each sample, samples were vortexed and centrifuged at 13,000 RPM for 10 min. Approximately 500 μl of supernatant was collected and 2× as much ethanol was added to precipitate the DNA. The mixture was centrifuged, the supernatant discarded and DNA pellet was re-suspended in 50 μl of TE buffer. PCR reaction was set up using the following primers: **5–8Δ F**, GCCGGCCAGGCAGCTCTTAC, and **5–8 Δ R**, GGCCAGTCACCATGGCCAGC, provide a 335 bp product only when RelA^CKO^ allele undergoes recombination and **7–8 F**, ACACTGCCGAGCTCAAGATC, and **7–8 R**, AGCTGCATGGAGACTCGAAC, provide a 425 bp product when WT RelA is present. Presence of RelA^CKO^ alleles was determined by **RelAFlx F**, TGCAAACAGACCTCCTTTGTCTTGA, and **RelAFlx R**, TCCTGAGACCAGACTCCTCCTCC, primers which provides a 450 bp product if the CKO allele is present or 270 bp product for a WT allele. PCR reaction was denatured at 94 °C for 2 min and subjected to 35 cycles of 30 s at 94 °C, 30 s at 54 °C, and 60 s at 72 °C. The PCR products were separated on a 1 % agarose gel containing ethidium bromide and imaged on a UV transilliumintor. Col1a2-CreER mice were genotyped according to instructions by The Jackson Laboratory (stock no. 016237).

### Quantitative RT-PCR (QRT-PCR)

RNA was extracted from MEF cells using Trizol Reagent (Life Technologies, 15596-026) and was quantified using NanoDrop 2000 (Thermo Scientific). 1 μg RNA was reverse transcribed to cDNA using SuperScript III First-Strand Synthesis System in a 20 μl reaction according to the manufacturer’s instruction (Invitrogen, 18080-51). cDNA was diluted 1:2 and 3 μl of the product was used in a 30 μl reaction mixture containing 15 μl SybrGreen mix and 500 nM final concentration of RelA forward, CCGGGATGGCTACTATGAGG, and RelA reverse, TCTTCACACACTGGATCCCC, primers or 18s rRNA forward, AGTCCCTGCCCTTTGTACACA, and 18s rRNA reverse, CGATCCGAGGGCCTCACTA, primers. The reaction mixtures were aliquoted into a Bio-Rad 96-well PCR plate and sealed. The plates were denatured at 95 °C for 3 min followed by 40 cycles of 15 s at 95 °C, 60 s at 60 °C and 1 min at 72 °C. PCR products were subjected to melting curve analysis to ensure that a single product was produced. Change in gene expression was determined using ΔΔCT method. RelA mRNA was normalized to 18s rRNA.

### Immunohistochemistry

Mouse embryos were formalin fixed and paraffin embedded. Tissue sections (6 μm) were deparaffinized and rehydrated; antigen retrieval was performed with 10 mM Sodium Citrate, pH6 before blocking with 5 % goat serum and incubation with anti-RelA C-terminus (Santa Cruz sc-3702, 1:200, or Abcam ab7970, 1:300), anti-RelA N-terminus (Santa Cruz sc-109, 1:100), anti-cleaved capase 3 (Cell Signaling 5A1E, 1:100) or anti-cleaved PARP antibody (Cell Signaling D64E10, 1:50) overnight at 4 °C. A biotinylated secondary antibody and avidin-biotin complex (Vector Labs, PK6101) was used to amplify the signal and DAB substrate (Vector Labs, SK4100) was used to detect the antigen-antibody complex. Images were obtained at 600× magnification using a Nikon digital camera DXM1200F attached to a Nikon Eclipse 80i microscope.

### Semiquantitative Western blot

Whole cell protein lysates were extracted from MEF cells using RIPA buffer containing protease inhibitor cocktail. The resultant protein mixture was fractionated by 10 % SDS-polyacrylamide gel electrophoresis and transferred to a PVDF membrane. After blocking with 5 % milk in TBS-tween buffer, the membrane was incubated with rabbit anti-RelA antibody (Santa Cruz sc-372, 1:1000) and mouse anti-beta actin (Sigma A5316, 1:10,000) overnight at 4 °C. Membranes were washed with TBS-tween before being incubated with IRDye 800-conjugated anti-rabbit and IRDye 700 conjugated anti-mouse secondary antibodies. RelA and beta-actin bands were detected using Licor Odyssey infrared scanner. Band densities were quantified using Image J (NIH) and represented in arbitrary units.

### Tamoxifen mediated genetic recombination and fibroblast culture

RelA^CKO/CKO^ female mice were crossed with Col1α2-CreER male mice (gift from Dr. Arjun Deb, UNC Chapel Hill), both on C57Bl/6 background, for three generations to generate RelA^CKO/CKO^ Cre + and RelA^CKO/CKO^ Cre- animals. Adult male Cre + and Cre- mice, 3–4 weeks old, were injected with Tamoxifen (Sigma T5648) 1 mg/day intraperitoneally for 10 days. Tamoxifen was dissolved in 10 % ethanol and 90 % corn oil for 10 mg/ml working solution. After another 3 weeks, mice were euthanized and tissues were harvested for characterization. Skin samples were incubated with 0.25 % trypsin overnight at 4 °C. The skin was then minced and digested in 0.14 Wunsch units/ml Liberase Blendzyme 3 (Roche) containing 1× antibiotic/antimycotic (Invitrogen) in DMEM/F12 media for 1–2 h at 37 °C. The skin was further dissociated and resuspended in DMEM/12 media containing 10 % FBS, 1× antibiotic/antimycotic and nonessential amino acids (complete media). Cells were centrifuged at 1000×g for 10 mins and the pellet was resuspended in complete media for culturing. After 3 passages, only surviving cells were dermal fibroblasts which were characterized via immunofluorescence, QRT-PCR and Western blot.

### Statistical analysis

Student’s t-test (2-tail, assuming unequal variance) was use to analyze difference between two groups. *P* < 0.05 was considered statistically significant.

## Additional file

Additional file 1: Figure S1. Whole cell lysates of A549 cells were prepared by hypotonic lysis in 50 mM NaCl, 10 mM Tris, pH 7.8, 2 mM EDTA, 1 % IGEPALCA-630, with protease inhibitor cocktail. Equal volumes of whole cell lysates were immunoprecipitated overnight with 4 μg of indicated antibody. Immunoprecipitates were captured on protein A magnetic beads and washed 4× in PBS. Samples were subjected to on-bead digestion and assayed for RelA using SID-SRM-MS [24]. Shown is mean +/- SD of RelA 756 signal relative to internal stable isotope standard (SIS). Note significant enrichment of RelA signal with each antibody. (PSD 150 kb)

## Abbreviations

CKO: Conditional knock-out
NF-kB: Nuclear factor-kappa B
RHD: Rel homology domain
TAD: Transcription activation domain
